# (1,10-Phenanthroline-κ^2^ *N*,*N*′)(triphenyl­phosphine-κ*P*)silver(I) trifluoro­methane­sulfonate

**DOI:** 10.1107/S1600536809032097

**Published:** 2009-08-19

**Authors:** Jie-Qiang Wu, Qiong-Hua Jin, Ke-Yi Hu, Cun-Lin Zhang

**Affiliations:** a Department of Chemistry, Capital Normal University, Beijing 100048, People’s Republic of China; b Department of Physics, Capital Normal University, Beijing 100048, People’s Republic of China

## Abstract

The structure of the title complex, [Ag(C_12_H_8_N_2_)(C_18_H_15_P)]CF_3_SO_3_, is based on a distorted trigonal–planar N_2_P coordination of the Ag^I^ ion, provided by two N atoms of the bidentate phenanthroline ligand and one P atom of the triphenyl­phosphine ligand. The phenanthroline ligand and one phenyl ring of the triphenyl­phosphine ligand almost lie in one plane (maximum deviation = 0.014 Å from the best planes). The crystal structure may be stabilized by an inter­molecular C—H⋯O hydrogen bond between the phenanthroline ligand and the O atom of the trifluoro­methane­sulfonate anion.

## Related literature

For related structures, see: Di Nicola *et al.* (2007 ▶); Jin *et al.* (1999 ▶, 2009 ▶); Effendy *et al.* (2007*a* ▶,*b* ▶); Awaleh *et al.* (2005*a* ▶,*b* ▶); Pettinari *et al.* (2007 ▶). For general background, see: Howells & Mccown (1977 ▶); Bowmaker *et al.* (2005 ▶); Lawrance (1986 ▶).
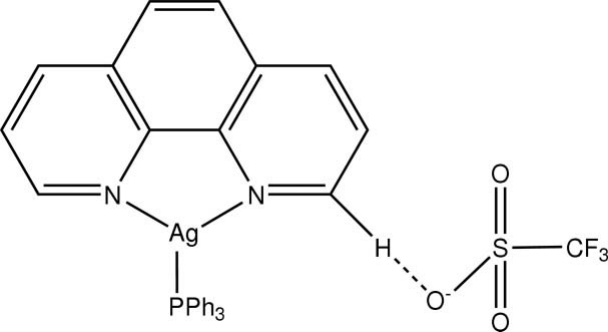


## Experimental

### 

#### Crystal data

[Ag(C_12_H_8_N_2_)(C_18_H_15_P)]CF_3_SO_3_
*M* *_r_* = 699.42
Triclinic, 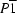

*a* = 10.9832 (2) Å
*b* = 11.7533 (2) Å
*c* = 12.2642 (3) Å
α = 77.711 (1)°
β = 76.183 (1)°
γ = 73.440 (1)°
*V* = 1455.66 (5) Å^3^
*Z* = 2
Mo *K*α radiation
μ = 0.87 mm^−1^
*T* = 293 K
0.4 × 0.3 × 0.2 mm

#### Data collection

Bruker SMART CCD area-detector diffractometer
Absorption correction: multi-scan (*SADABS*; Bruker, 2007 ▶) *T*_min_ = 0.735, *T*_max_ = 0.832
18629 measured reflections
9515 independent reflections
6777 reflections with *I* > 2σ(*I*)
*R*_int_ = 0.021

#### Refinement

*R*[*F* ^2^ > 2σ(*F* ^2^)] = 0.042
*wR*(*F* ^2^) = 0.133
*S* = 1.00
9515 reflections
379 parameters
H-atom parameters constrained
Δρ_max_ = 0.59 e Å^−3^
Δρ_min_ = −0.66 e Å^−3^

### 

Data collection: *SMART* (Bruker, 2007 ▶); cell refinement: *SAINT-Plus* (Bruker, 2007 ▶); data reduction: *SAINT-Plus*; program(s) used to solve structure: *SHELXS97* (Sheldrick, 2008 ▶); program(s) used to refine structure: *SHELXL97* (Sheldrick, 2008 ▶); molecular graphics: *SHELXTL* (Sheldrick, 2008 ▶); software used to prepare material for publication: *SHELXTL*.

## Supplementary Material

Crystal structure: contains datablocks global, I. DOI: 10.1107/S1600536809032097/wm2241sup1.cif

Structure factors: contains datablocks I. DOI: 10.1107/S1600536809032097/wm2241Isup2.hkl

Additional supplementary materials:  crystallographic information; 3D view; checkCIF report

## Figures and Tables

**Table:** 

Ag1—N2	2.2798 (18)
Ag1—N1	2.292 (2)
Ag1—P1	2.3469 (5)

**Table:** 

N2—Ag1—N1	73.53 (8)
N2—Ag1—P1	147.77 (6)
N1—Ag1—P1	138.03 (6)

**Table 2:** Hydrogen-bond geometry (Å, °)

*D*—H⋯*A*	*D*—H	H⋯*A*	*D*⋯*A*	*D*—H⋯*A*
C20—H16⋯O2	0.93	2.36	3.285 (6)	173

**Fig. 1.**
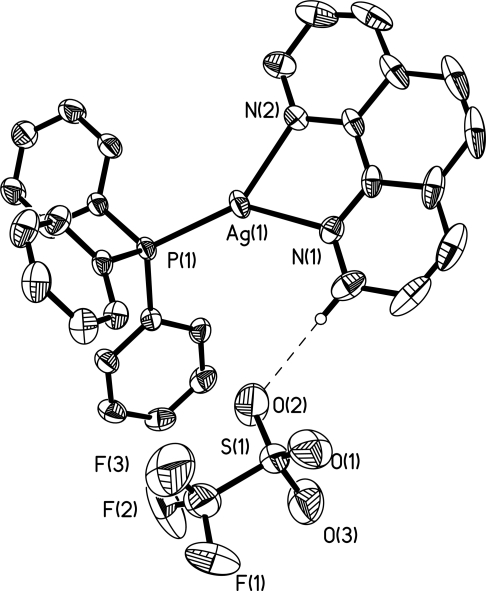
Perspective view of the molecule of the title complex; hydrogen atoms are omitted for clarity. Atoms are displayed as ellipsoids at the 35% probability level.

## supplementary crystallographic information

### Comment

A recent report (Di Nicola *et al.*, 2007) describes complexes between silver nitrate, a tertiary phosphine ligand and oligodentate bases, *L* that are derivatives of 2,2'-bipyridyl, which resulted in adducts with general formula AgNO_3_:P*R*_3_:*L*(1:1:1). The silver coordination environment in these complexes is dominated by the quasi-planar N_2_AgP or O_2_AgP coordination. We have likewise studied mixed-ligand Ag(I) complexes of *N-*heterocyclic and PPh_3_ ligands, *viz* [AgBr(phen)(PPh_3_)] and [Ag*X*(2-Apy)(PPh_3_)]_2_ (*X* = Br, Cl, NO_3_; 2-Apy= 2-aminopyridine) (Jin *et al.*, 1999, Jin *et al.*, 2009) and have synthesized the title complex [Ag(phen)(PPh_3_)](OTf). Furthermore, we have studied the role of several weakly coordinating anions (nitrate, nitrite, acetate, perchlorate trifluoroacetate and trifluoromethanesulfonate) in silver complexes.

The molecular structure of the title complex is depicted in Fig.1. The coordination polyhedron of the silver atom adopts a distorted trigonal-planar geometry, formed by two nitrogen atoms of phen with Ag—N distances of 2.3469 (5) Å and 2.2797 (19) Å, and by one phosphorus atom of the PPh_3_ ligand with a Ag—P distance of 2.292 (2) Å. The trifluoromethanesulfonate anion is present as a counter anion and, as expected, shows no direct coordination to the metal center, in contrast to the complex [AgBr(phen)(PPh_3_)] where the silver atom is coordinated to two nitrogen atoms of phen (Ag—N 2.376 (8) Å), one phosphorus atom of PPh_3_ (Ag—P, 2.375 (3) Å) and in addition to one bromide anion (Jin *et al.*, 1999), adopting a distorted tetrahedron as coordination polyhedron.

The molecular structure of the title complex shows little differences in comparison with the structures of compounds AgX:PPh_3_:*L*, where *X* = nitrate (Di Nicola *et al.*, 2007), nitrite (Pettinari *et al.*, 2007), acetate (Effendy *et al.*, 2007*a*), perchlorate (Effendy *et al.*, 2007*b*) and trifluoroacetate (Awaleh *et al.*, 2005*a*). Considering the large steric hindrance and the weak coordination ability (Awaleh *et al.*, 2005*b*; Howells *et al.*, 1977; Lawrance *et al.*,1986) of the trifluoromethanesulfonate anion, there is only one C—H···O hydrogen-bond between the phenanthroline ligand and the O atom of the anion with the distance O···H of 2.609Å and the angle C—H···O of 173°.

In the title complex, the P—Ag—N1, P—Ag—N2 and N1—Ag—N2 angles are 147.77 (6)°, 138.03 (6)° and 73.54 (8) ° with a sum of 359.54 °, which comfirms the trigonal-planar environment around the silver atom. In the silver nitrate complex, the P—Ag—N (132.66 (9)°, 131.76 (8)°) (Di Nicola *et al.*, 2007) angles are similar. However, contributing to the role of the nitrate anion, the coordination environment of silver changes from distorted trigonal planar to tetrahedral. The P—Ag—N angles in the other complexes are: 136.94 (5)°, 139.60 (5)°, 71.40 (6)° in the perchlorate (Effendy *et al.*, 2007*b*), 129.4 (1)°, 135.7 (1)°, 71.7 (2)° in trifluoroacetate (Awaleh *et al.*, 2005*a*), 116.52 (6)°, 126.12 (7)°, 70.5 (1)° in acetate (Effendy *et al.*, 2007*a*) and 126.72 (8)°, 127.18 (9)°, 70.77 (12)° in the nitrite (Pettinari *et al.*,2007) anion.

Hence, we should consider two types of anions in the complexes AgX:P*R*_3_:*L*, *viz* tetrahedral or distorted trigonal-planar anions and planar or quasi-planar anions (Awaleh *et al.*, 2005*a*; Awaleh *et al.*, 2005*b*; Bowmaker *et al.*, 2005). Nitrate, nitrite and acetate belong to the former type, whereas perchlorate, trifluoroacetate and trifluoromethanesulfonate can play a role in both of them because of large steric hindrance and the weak coordination ability.

### Experimental

A mixture of AgOTf, Ph_3_P and phen in the molar ratio of 1:1:1 in MeOH was stirred for 1 h at ambient temperature, then filtered. Subsequent slow evaporation of the filtrate resulted in the formation of colorless crystals of the title complex. Crystals suitable for single-crystal X-ray diffraction were selected directly from the sample as prepared. Analysis found (percentage): C 53.22, H 3.29, N 4.01; calculated: C 53.19, H 3.29, N 4.02.

### Refinement

All hydrogen atoms were located in the calculated sites and included in the final refinement in the riding model approximation with displacement parameters derived from the parent atoms to which they were bonded (U_eq_(H) = 1.2U_eq_(C)).

### Crystal data

**Table:**

[Ag(C_12_H_8_N_2_)(C_18_H_15_P)]CF_3_SO_3_	*Z* = 2
*M**_r_* = 699.42	*F*(000) = 704
Triclinic, *P*1	*D*_x_ = 1.596 Mg m^−^^3^
Hall symbol: -P 1	Mo *K*α radiation, λ = 0.71073 Å
*a* = 10.9832 (2) Å	Cell parameters from 5045 reflections
*b* = 11.7533 (2) Å	θ = 2.3–32.9°
*c* = 12.2642 (3) Å	µ = 0.87 mm^−^^1^
α = 77.711 (1)°	*T* = 293 K
β = 76.183 (1)°	Block, colourless
γ = 73.440 (1)°	0.4 × 0.3 × 0.2 mm
*V* = 1455.66 (5) Å^3^	

### Data collection

**Table:**

Bruker SMART CCD area-detector diffractometer	9515 independent reflections
Radiation source: fine-focus sealed tube	6777 reflections with *I* > 2σ(*I*)
graphite	*R*_int_ = 0.021
φ and ω scans	θ_max_ = 32.6°, θ_min_ = 2.0°
Absorption correction: multi-scan (*SADABS*; Bruker, 2007)	*h* = −15→15
*T*_min_ = 0.735, *T*_max_ = 0.832	*k* = −17→16
18629 measured reflections	*l* = −18→16

### Refinement

**Table:**

Refinement on *F*^2^	Primary atom site location: structure-invariant direct methods
Least-squares matrix: full	Secondary atom site location: difference Fourier map
*R*[*F*^2^ > 2σ(*F*^2^)] = 0.042	Hydrogen site location: inferred from neighbouring sites
*wR*(*F*^2^) = 0.133	H-atom parameters constrained
*S* = 1.00	*w* = 1/[σ^2^(*F*_o_^2^) + (0.075*P*)^2^ + 0.38*P*] where *P* = (*F*_o_^2^ + 2*F*_c_^2^)/3
9515 reflections	(Δ/σ)_max_ = 0.001
379 parameters	Δρ_max_ = 0.59 e Å^−^^3^
0 restraints	Δρ_min_ = −0.66 e Å^−^^3^

### Special details

**Table:**

Geometry. All e.s.d.'s (except the e.s.d. in the dihedral angle between two l.s. planes) are estimated using the full covariance matrix. The cell e.s.d.'s are taken into account individually in the estimation of e.s.d.'s in distances, angles and torsion angles; correlations between e.s.d.'s in cell parameters are only used when they are defined by crystal symmetry. An approximate (isotropic) treatment of cell e.s.d.'s is used for estimating e.s.d.'s involving l.s. planes.
Refinement. Refinement of *F*^2^ against ALL reflections. The weighted *R*-factor *wR* and goodness of fit *S* are based on *F*^2^, conventional *R*-factors *R* are based on *F*, with *F* set to zero for negative *F*^2^. The threshold expression of *F*^2^ > σ(*F*^2^) is used only for calculating *R*-factors(gt) *etc*. and is not relevant to the choice of reflections for refinement. *R*-factors based on *F*^2^ are statistically about twice as large as those based on *F*, and *R*- factors based on ALL data will be even larger.

### Fractional atomic coordinates and isotropic or equivalent isotropic displacement parameters (Å^2^)

**Table:**

	*x*	*y*	*z*	*U*_iso_*/*U*_eq_	
Ag1	0.09032 (2)	0.931304 (15)	0.211327 (16)	0.05664 (9)	
P1	0.18488 (6)	0.77307 (5)	0.10540 (5)	0.04241 (13)	
C24	−0.0779 (3)	1.12309 (19)	0.36019 (18)	0.0491 (6)	
C25	−0.3066 (4)	1.2209 (4)	0.3869 (4)	0.0981 (15)	
H21	−0.3787	1.2746	0.4205	0.118*	
S1	0.61400 (9)	0.77152 (8)	0.31032 (8)	0.0748 (2)	
C1	0.0853 (2)	0.75012 (19)	0.01682 (19)	0.0455 (5)	
C3	0.1327 (3)	0.6742 (3)	−0.0638 (3)	0.0631 (7)	
H6	0.2207	0.6372	−0.0786	0.076*	
C2	−0.0452 (3)	0.8068 (3)	0.0342 (3)	0.0644 (7)	
H10	−0.0782	0.8614	0.0852	0.077*	
C4	0.3400 (2)	0.77960 (19)	0.01460 (18)	0.0433 (4)	
C6	0.4324 (3)	0.6788 (2)	−0.0199 (2)	0.0566 (6)	
H5	0.4166	0.6031	0.0064	0.068*	
C7	0.4818 (3)	0.9018 (3)	−0.0982 (3)	0.0707 (8)	
H2	0.4979	0.9772	−0.1261	0.085*	
C5	0.3678 (3)	0.8918 (2)	−0.0246 (2)	0.0562 (6)	
H1	0.3088	0.9603	−0.0008	0.067*	
C10	0.1297 (3)	0.5596 (3)	0.2319 (2)	0.0602 (7)	
H15	0.0575	0.5801	0.1981	0.072*	
C8	0.2170 (2)	0.63074 (19)	0.20106 (18)	0.0438 (5)	
C11	0.1495 (4)	0.4583 (3)	0.3128 (3)	0.0778 (10)	
H14	0.0894	0.4118	0.3342	0.093*	
C9	0.3237 (3)	0.5981 (2)	0.2526 (2)	0.0544 (6)	
H11	0.3828	0.6456	0.2335	0.065*	
C12	0.5478 (3)	0.6894 (3)	−0.0929 (3)	0.0652 (7)	
H4	0.6082	0.6213	−0.1158	0.078*	
C13	0.5725 (3)	0.8001 (3)	−0.1309 (3)	0.0677 (8)	
H3	0.6504	0.8071	−0.1789	0.081*	
C14	−0.1276 (3)	0.7833 (3)	−0.0234 (3)	0.0766 (9)	
H9	−0.2156	0.8201	−0.0094	0.092*	
C16	0.0513 (3)	0.6529 (3)	−0.1223 (3)	0.0715 (8)	
H7	0.0846	0.6022	−0.1768	0.086*	
C15	−0.0786 (3)	0.7058 (3)	−0.1007 (3)	0.0714 (8)	
H8	−0.1336	0.6889	−0.1388	0.086*	
C18	0.3430 (4)	0.4952 (3)	0.3324 (3)	0.0685 (8)	
H12	0.4153	0.4733	0.3662	0.082*	
C17	0.2555 (4)	0.4256 (3)	0.3616 (3)	0.0763 (9)	
H13	0.2688	0.3561	0.4148	0.092*	
O1	0.5839 (4)	0.7776 (4)	0.4276 (3)	0.1266 (12)	
O2	0.5105 (4)	0.7955 (3)	0.2518 (4)	0.1519 (17)	
O3	0.7108 (4)	0.8336 (4)	0.2534 (4)	0.1455 (16)	
C19	0.6933 (5)	0.6175 (4)	0.2985 (6)	0.1103 (17)	
F1	0.7991 (3)	0.5804 (4)	0.3393 (4)	0.1752 (17)	
F2	0.7172 (6)	0.5954 (4)	0.1989 (4)	0.218 (3)	
C23	0.0474 (3)	1.1023 (2)	0.38492 (18)	0.0513 (6)	
C21	0.0633 (4)	1.1640 (3)	0.4660 (2)	0.0763 (11)	
C22	−0.0480 (7)	1.2472 (3)	0.5189 (3)	0.1041 (18)	
H19	−0.0385	1.2879	0.5723	0.125*	
C20	0.2638 (4)	1.0021 (3)	0.3561 (3)	0.0752 (9)	
H16	0.3327	0.9473	0.3203	0.090*	
N1	0.1478 (2)	1.02361 (18)	0.33127 (18)	0.0522 (5)	
N2	−0.09368 (19)	1.06137 (17)	0.28528 (15)	0.0489 (5)	
C26	−0.1838 (4)	1.2062 (2)	0.4146 (2)	0.0757 (10)	
C27	−0.2110 (3)	1.0794 (3)	0.2637 (3)	0.0708 (8)	
H23	−0.2217	1.0367	0.2123	0.085*	
C28	−0.1636 (6)	1.2669 (3)	0.4930 (3)	0.1029 (17)	
H20	−0.2329	1.3223	0.5277	0.124*	
C29	−0.3190 (4)	1.1595 (4)	0.3146 (4)	0.0990 (14)	
H22	−0.3996	1.1693	0.2972	0.119*	
C31	0.2848 (6)	1.0623 (5)	0.4377 (4)	0.1018 (17)	
H17	0.3670	1.0475	0.4536	0.122*	
C30	0.1856 (6)	1.1398 (5)	0.4908 (3)	0.1008 (17)	
H18	0.1988	1.1777	0.5448	0.121*	
F3	0.6223 (5)	0.5475 (3)	0.3633 (5)	0.213 (3)	

### Atomic displacement parameters (Å^2^)

**Table:**

	*U*^11^	*U*^22^	*U*^33^	*U*^12^	*U*^13^	*U*^23^
Ag1	0.06687 (15)	0.04557 (11)	0.05563 (12)	−0.00722 (8)	−0.00064 (9)	−0.02543 (8)
P1	0.0462 (3)	0.0368 (2)	0.0428 (3)	−0.0074 (2)	−0.0011 (2)	−0.0152 (2)
C24	0.0684 (16)	0.0344 (9)	0.0358 (9)	−0.0065 (9)	0.0011 (9)	−0.0071 (7)
C25	0.072 (2)	0.074 (2)	0.094 (3)	0.0230 (17)	0.021 (2)	0.0052 (19)
S1	0.0677 (5)	0.0771 (5)	0.0829 (5)	−0.0120 (4)	−0.0169 (4)	−0.0249 (4)
C1	0.0490 (13)	0.0417 (10)	0.0443 (11)	−0.0082 (8)	−0.0041 (9)	−0.0133 (8)
C3	0.0536 (15)	0.0689 (16)	0.0681 (16)	−0.0009 (12)	−0.0068 (12)	−0.0380 (13)
C2	0.0545 (16)	0.0662 (16)	0.0732 (18)	−0.0011 (12)	−0.0090 (13)	−0.0345 (13)
C4	0.0472 (12)	0.0395 (9)	0.0426 (10)	−0.0110 (8)	−0.0056 (9)	−0.0077 (8)
C6	0.0523 (15)	0.0434 (11)	0.0633 (15)	−0.0094 (10)	0.0063 (11)	−0.0083 (10)
C7	0.075 (2)	0.0628 (16)	0.077 (2)	−0.0352 (15)	−0.0136 (16)	0.0069 (14)
C5	0.0613 (16)	0.0413 (11)	0.0690 (16)	−0.0161 (10)	−0.0162 (13)	−0.0062 (10)
C10	0.0700 (18)	0.0622 (15)	0.0540 (14)	−0.0303 (13)	−0.0077 (12)	−0.0061 (11)
C8	0.0538 (13)	0.0407 (9)	0.0377 (9)	−0.0145 (9)	−0.0004 (9)	−0.0133 (8)
C11	0.107 (3)	0.0724 (19)	0.0640 (18)	−0.0527 (19)	−0.0103 (18)	0.0020 (15)
C9	0.0615 (16)	0.0525 (12)	0.0524 (13)	−0.0168 (11)	−0.0113 (11)	−0.0106 (10)
C12	0.0510 (15)	0.0647 (16)	0.0652 (16)	−0.0060 (12)	0.0046 (12)	−0.0074 (13)
C13	0.0497 (16)	0.082 (2)	0.0651 (17)	−0.0229 (14)	−0.0056 (13)	0.0055 (14)
C14	0.0515 (17)	0.086 (2)	0.098 (2)	0.0036 (14)	−0.0254 (16)	−0.0410 (18)
C16	0.071 (2)	0.0799 (19)	0.0713 (18)	−0.0068 (15)	−0.0163 (15)	−0.0411 (15)
C15	0.071 (2)	0.0742 (18)	0.077 (2)	−0.0096 (15)	−0.0304 (16)	−0.0228 (15)
C18	0.093 (2)	0.0581 (15)	0.0577 (15)	−0.0160 (15)	−0.0278 (15)	−0.0039 (12)
C17	0.118 (3)	0.0585 (16)	0.0554 (16)	−0.0331 (17)	−0.0203 (17)	0.0037 (12)
O1	0.105 (2)	0.169 (4)	0.097 (2)	−0.019 (2)	0.0043 (18)	−0.050 (2)
O2	0.161 (3)	0.090 (2)	0.238 (5)	0.013 (2)	−0.143 (4)	−0.038 (2)
O3	0.148 (3)	0.117 (3)	0.172 (4)	−0.072 (3)	0.034 (3)	−0.043 (2)
C19	0.094 (3)	0.070 (2)	0.161 (5)	−0.011 (2)	−0.041 (3)	0.002 (3)
F1	0.092 (2)	0.166 (3)	0.236 (4)	0.038 (2)	−0.057 (2)	−0.031 (3)
F2	0.336 (7)	0.123 (3)	0.175 (4)	0.056 (3)	−0.082 (4)	−0.096 (3)
C23	0.0848 (19)	0.0382 (10)	0.0355 (10)	−0.0281 (11)	−0.0072 (10)	−0.0027 (8)
C21	0.146 (3)	0.0638 (16)	0.0408 (12)	−0.068 (2)	−0.0155 (16)	0.0002 (11)
C22	0.216 (6)	0.0626 (19)	0.0432 (15)	−0.068 (3)	0.011 (2)	−0.0225 (13)
C20	0.071 (2)	0.080 (2)	0.080 (2)	−0.0389 (17)	−0.0279 (17)	0.0172 (16)
N1	0.0608 (13)	0.0474 (10)	0.0525 (11)	−0.0202 (9)	−0.0160 (9)	−0.0014 (8)
N2	0.0510 (12)	0.0468 (10)	0.0441 (10)	−0.0026 (8)	−0.0093 (8)	−0.0096 (8)
C26	0.104 (3)	0.0428 (12)	0.0515 (14)	0.0004 (13)	0.0185 (15)	−0.0092 (10)
C27	0.0586 (18)	0.0790 (19)	0.0655 (17)	−0.0017 (14)	−0.0208 (14)	−0.0024 (14)
C28	0.173 (5)	0.0529 (16)	0.061 (2)	−0.023 (2)	0.029 (3)	−0.0266 (14)
C29	0.060 (2)	0.108 (3)	0.093 (3)	0.011 (2)	−0.0072 (19)	0.009 (2)
C31	0.120 (4)	0.131 (4)	0.089 (3)	−0.094 (3)	−0.059 (3)	0.040 (3)
C30	0.171 (5)	0.109 (3)	0.061 (2)	−0.103 (4)	−0.035 (3)	0.011 (2)
F3	0.209 (5)	0.103 (2)	0.335 (7)	−0.079 (3)	−0.087 (4)	0.042 (3)

### Geometric parameters (Å, °)

**Table:**

Ag1—N2	2.2798 (18)	C11—H14	0.9300
Ag1—N1	2.292 (2)	C9—C18	1.384 (4)
Ag1—P1	2.3469 (5)	C9—H11	0.9300
P1—C4	1.812 (2)	C12—C13	1.365 (4)
P1—C1	1.819 (3)	C12—H4	0.9300
P1—C8	1.823 (2)	C13—H3	0.9300
C24—N2	1.353 (3)	C14—C15	1.364 (5)
C24—C26	1.418 (3)	C14—H9	0.9300
C24—C23	1.422 (4)	C16—C15	1.370 (5)
C25—C29	1.308 (7)	C16—H7	0.9300
C25—C26	1.423 (7)	C15—H8	0.9300
C25—H21	0.9300	C18—C17	1.371 (5)
S1—O1	1.409 (3)	C18—H12	0.9300
S1—O2	1.417 (3)	C17—H13	0.9300
S1—O3	1.421 (4)	C19—F2	1.253 (7)
S1—C19	1.791 (5)	C19—F1	1.298 (6)
C1—C3	1.381 (3)	C19—F3	1.307 (6)
C1—C2	1.384 (4)	C23—N1	1.355 (3)
C3—C16	1.375 (4)	C23—C21	1.416 (3)
C3—H6	0.9300	C21—C30	1.385 (7)
C2—C14	1.387 (5)	C21—C22	1.442 (7)
C2—H10	0.9300	C22—C28	1.326 (7)
C4—C6	1.391 (3)	C22—H19	0.9300
C4—C5	1.397 (3)	C20—N1	1.323 (4)
C6—C12	1.387 (4)	C20—C31	1.435 (6)
C6—H5	0.9300	C20—H16	0.9300
C7—C5	1.376 (4)	N2—C27	1.327 (4)
C7—C13	1.385 (5)	C26—C28	1.403 (6)
C7—H2	0.9300	C27—C29	1.394 (5)
C5—H1	0.9300	C27—H23	0.9300
C10—C11	1.380 (4)	C28—H20	0.9300
C10—C8	1.382 (4)	C29—H22	0.9300
C10—H15	0.9300	C31—C30	1.337 (7)
C8—C9	1.384 (4)	C31—H17	0.9300
C11—C17	1.356 (5)	C30—H18	0.9300
			
N2—Ag1—N1	73.53 (8)	C7—C13—H3	119.9
N2—Ag1—P1	147.77 (6)	C15—C14—C2	119.6 (3)
N1—Ag1—P1	138.03 (6)	C15—C14—H9	120.2
C4—P1—C1	106.47 (11)	C2—C14—H9	120.2
C4—P1—C8	104.71 (10)	C15—C16—C3	120.4 (3)
C1—P1—C8	103.84 (10)	C15—C16—H7	119.8
C4—P1—Ag1	115.16 (7)	C3—C16—H7	119.8
C1—P1—Ag1	115.70 (7)	C14—C15—C16	120.1 (3)
C8—P1—Ag1	109.82 (7)	C14—C15—H8	119.9
N2—C24—C26	121.2 (3)	C16—C15—H8	119.9
N2—C24—C23	118.8 (2)	C17—C18—C9	120.1 (3)
C26—C24—C23	120.0 (3)	C17—C18—H12	120.0
C29—C25—C26	120.5 (3)	C9—C18—H12	120.0
C29—C25—H21	119.7	C11—C17—C18	119.9 (3)
C26—C25—H21	119.7	C11—C17—H13	120.0
O1—S1—O2	118.2 (3)	C18—C17—H13	120.0
O1—S1—O3	111.0 (3)	F2—C19—F1	109.1 (6)
O2—S1—O3	113.3 (3)	F2—C19—F3	108.5 (6)
O1—S1—C19	105.7 (3)	F1—C19—F3	102.2 (5)
O2—S1—C19	103.3 (2)	F2—C19—S1	113.4 (4)
O3—S1—C19	103.5 (3)	F1—C19—S1	112.9 (4)
C3—C1—C2	118.2 (3)	F3—C19—S1	110.1 (4)
C3—C1—P1	123.0 (2)	N1—C23—C21	121.9 (3)
C2—C1—P1	118.73 (18)	N1—C23—C24	119.3 (2)
C16—C3—C1	120.6 (3)	C21—C23—C24	118.8 (3)
C16—C3—H6	119.7	C30—C21—C23	117.7 (4)
C1—C3—H6	119.7	C30—C21—C22	123.6 (4)
C1—C2—C14	121.0 (3)	C23—C21—C22	118.7 (4)
C1—C2—H10	119.5	C28—C22—C21	121.5 (3)
C14—C2—H10	119.5	C28—C22—H19	119.2
C6—C4—C5	118.1 (2)	C21—C22—H19	119.2
C6—C4—P1	123.43 (18)	N1—C20—C31	120.8 (4)
C5—C4—P1	118.47 (19)	N1—C20—H16	119.6
C12—C6—C4	121.0 (2)	C31—C20—H16	119.6
C12—C6—H5	119.5	C20—N1—C23	119.3 (3)
C4—C6—H5	119.5	C20—N1—Ag1	126.7 (2)
C5—C7—C13	120.2 (3)	C23—N1—Ag1	113.81 (17)
C5—C7—H2	119.9	C27—N2—C24	118.7 (2)
C13—C7—H2	119.9	C27—N2—Ag1	126.7 (2)
C7—C5—C4	120.5 (3)	C24—N2—Ag1	114.57 (16)
C7—C5—H1	119.7	C28—C26—C24	119.4 (4)
C4—C5—H1	119.7	C28—C26—C25	123.8 (4)
C11—C10—C8	120.1 (3)	C24—C26—C25	116.9 (3)
C11—C10—H15	120.0	N2—C27—C29	122.8 (4)
C8—C10—H15	120.0	N2—C27—H23	118.6
C10—C8—C9	118.8 (2)	C29—C27—H23	118.6
C10—C8—P1	121.0 (2)	C22—C28—C26	121.6 (4)
C9—C8—P1	119.95 (18)	C22—C28—H20	119.2
C17—C11—C10	120.8 (3)	C26—C28—H20	119.2
C17—C11—H14	119.6	C25—C29—C27	119.9 (4)
C10—C11—H14	119.6	C25—C29—H22	120.1
C8—C9—C18	120.3 (3)	C27—C29—H22	120.1
C8—C9—H11	119.9	C30—C31—C20	119.8 (4)
C18—C9—H11	119.9	C30—C31—H17	120.1
C13—C12—C6	119.9 (3)	C20—C31—H17	120.1
C13—C12—H4	120.1	C31—C30—C21	120.4 (4)
C6—C12—H4	120.1	C31—C30—H18	119.8
C12—C13—C7	120.2 (3)	C21—C30—H18	119.8
C12—C13—H3	119.9		
			
N2—Ag1—P1—C4	143.08 (12)	O1—S1—C19—F1	−60.6 (5)
N1—Ag1—P1—C4	−51.26 (12)	O2—S1—C19—F1	174.5 (5)
N2—Ag1—P1—C1	18.06 (13)	O3—S1—C19—F1	56.2 (5)
N1—Ag1—P1—C1	−176.27 (11)	O1—S1—C19—F3	52.9 (5)
N2—Ag1—P1—C8	−99.05 (13)	O2—S1—C19—F3	−71.9 (5)
N1—Ag1—P1—C8	66.61 (12)	O3—S1—C19—F3	169.7 (5)
C4—P1—C1—C3	40.1 (3)	N2—C24—C23—N1	1.4 (3)
C8—P1—C1—C3	−70.1 (3)	C26—C24—C23—N1	−179.3 (2)
Ag1—P1—C1—C3	169.5 (2)	N2—C24—C23—C21	−178.0 (2)
C4—P1—C1—C2	−142.9 (2)	C26—C24—C23—C21	1.3 (3)
C8—P1—C1—C2	106.9 (2)	N1—C23—C21—C30	−1.1 (4)
Ag1—P1—C1—C2	−13.6 (2)	C24—C23—C21—C30	178.3 (2)
C2—C1—C3—C16	−2.1 (5)	N1—C23—C21—C22	179.5 (2)
P1—C1—C3—C16	174.9 (3)	C24—C23—C21—C22	−1.1 (3)
C3—C1—C2—C14	3.3 (5)	C30—C21—C22—C28	−179.5 (3)
P1—C1—C2—C14	−173.8 (3)	C23—C21—C22—C28	−0.1 (5)
C1—P1—C4—C6	−75.7 (2)	C31—C20—N1—C23	−0.9 (4)
C8—P1—C4—C6	33.9 (2)	C31—C20—N1—Ag1	−176.6 (2)
Ag1—P1—C4—C6	154.6 (2)	C21—C23—N1—C20	1.0 (3)
C1—P1—C4—C5	103.2 (2)	C24—C23—N1—C20	−178.4 (2)
C8—P1—C4—C5	−147.2 (2)	C21—C23—N1—Ag1	177.19 (17)
Ag1—P1—C4—C5	−26.5 (2)	C24—C23—N1—Ag1	−2.2 (3)
C5—C4—C6—C12	−1.2 (4)	N2—Ag1—N1—C20	177.5 (2)
P1—C4—C6—C12	177.6 (2)	P1—Ag1—N1—C20	5.5 (3)
C13—C7—C5—C4	−2.4 (5)	N2—Ag1—N1—C23	1.63 (15)
C6—C4—C5—C7	2.1 (4)	P1—Ag1—N1—C23	−170.45 (11)
P1—C4—C5—C7	−176.8 (2)	C26—C24—N2—C27	−0.4 (3)
C11—C10—C8—C9	0.1 (4)	C23—C24—N2—C27	178.9 (2)
C11—C10—C8—P1	−173.6 (3)	C26—C24—N2—Ag1	−179.12 (18)
C4—P1—C8—C10	−140.7 (2)	C23—C24—N2—Ag1	0.2 (3)
C1—P1—C8—C10	−29.2 (2)	N1—Ag1—N2—C27	−179.6 (2)
Ag1—P1—C8—C10	95.1 (2)	P1—Ag1—N2—C27	−9.5 (3)
C4—P1—C8—C9	45.7 (2)	N1—Ag1—N2—C24	−0.94 (15)
C1—P1—C8—C9	157.17 (19)	P1—Ag1—N2—C24	169.11 (11)
Ag1—P1—C8—C9	−78.53 (19)	N2—C24—C26—C28	179.0 (3)
C8—C10—C11—C17	−1.3 (5)	C23—C24—C26—C28	−0.3 (4)
C10—C8—C9—C18	0.8 (4)	N2—C24—C26—C25	0.2 (4)
P1—C8—C9—C18	174.6 (2)	C23—C24—C26—C25	−179.1 (2)
C4—C6—C12—C13	0.7 (5)	C29—C25—C26—C28	−178.6 (4)
C6—C12—C13—C7	−1.0 (5)	C29—C25—C26—C24	0.1 (5)
C5—C7—C13—C12	1.9 (5)	C24—N2—C27—C29	0.2 (4)
C1—C2—C14—C15	−1.9 (6)	Ag1—N2—C27—C29	178.8 (3)
C1—C3—C16—C15	−0.6 (6)	C21—C22—C28—C26	1.2 (6)
C2—C14—C15—C16	−0.9 (6)	C24—C26—C28—C22	−1.0 (5)
C3—C16—C15—C14	2.1 (6)	C25—C26—C28—C22	177.7 (3)
C8—C9—C18—C17	−0.6 (5)	C26—C25—C29—C27	−0.3 (6)
C10—C11—C17—C18	1.5 (6)	N2—C27—C29—C25	0.1 (6)
C9—C18—C17—C11	−0.5 (5)	N1—C20—C31—C30	1.1 (5)
O1—S1—C19—F2	174.6 (5)	C20—C31—C30—C21	−1.2 (5)
O2—S1—C19—F2	49.8 (6)	C23—C21—C30—C31	1.2 (5)
O3—S1—C19—F2	−68.6 (6)	C22—C21—C30—C31	−179.4 (3)

### Hydrogen-bond geometry (Å, °)

**Table:**

*D*—H···*A*	*D*—H	H···*A*	*D*···*A*	*D*—H···*A*
C20—H16···O2	0.93	2.36	3.285 (6)	173
